# Conductive Polymer-Based Electrodes and Supercapacitors: Materials, Electrolytes, and Characterizations

**DOI:** 10.3390/ma17164126

**Published:** 2024-08-20

**Authors:** Zahra Roohi, Frej Mighri, Ze Zhang

**Affiliations:** 1 Department of Chemical Engineering, Faculty of Sciences and Engineering, Université Laval, Quebec, QC G1V 0A6, Canada; zahra.roohi.1@ulaval.ca (Z.R.); frej.mighri@gch.ulaval.ca (F.M.); 2 Division of Regenerative Medicine, Saint-François d’Assise Hospital, Research Center of CHU de Québec—Université Laval, Quebec, QC G1L 3L5, Canada; 3 Department of Surgery, Faculty of Medicine, Université Laval, Quebec, QC G1V 0A6, Canada

**Keywords:** energy storage, conductive polymer, supercapacitor, electrochemistry, polypyrrole

## Abstract

New materials and the interactions between them are the basis of novel energy storage devices such as supercapacitors and batteries. In recent years, because of the increasing demand for electricity as an energy source, the development of new energy storage materials is among the most actively studied topics. Conductive polymers (CPs), because of their intrinsic electrochemical activity and electrical conductivity, have also been intensively explored. While most of the high capacitance reported in the literature comes from hybrid materials, for example, conductive polymers composed of metal oxides and carbon materials, such as graphene and carbon nanotubes, new chemistry and the 3D structure of conductive polymers remain critical. This comprehensive review focuses on the basic properties of three popular conductive polymers and their composites with carbon materials and metal oxides that have been actively explored as energy storage materials, i.e., polypyrrole (PPy), polyaniline (PANi), and polythiophene (PTh), and various types of electrolytes, including aqueous, organic, quasi-solid, and self-healing electrolytes. Important experimental parameters affecting material property and morphology are also discussed. Electrochemical and analytical techniques frequently employed in material and supercapacitor research are presented. In particular, cyclic voltammetry (CV) and electrochemical impedance spectroscopy (EIS) are discussed in detail, including how to extract data from spectra to calculate key parameters. Pros and cons of CP-based supercapacitors are discussed together with their potential applications.

## 1. Introduction

Global economic development and population growth increase fossil fuel consumption and pollution, forcing us to search for clean energy sources and new energy conversion and storage methods. Electrochemical energy is an essential part of the clean energy portfolio.

The most common electrochemical energy storage systems are batteries, fuel cells, and electrochemical supercapacitors (ESCs or SCs). Among these systems, SCs are characterized by their high power density, long life cycle, and fast charging.

**Figure 1:**
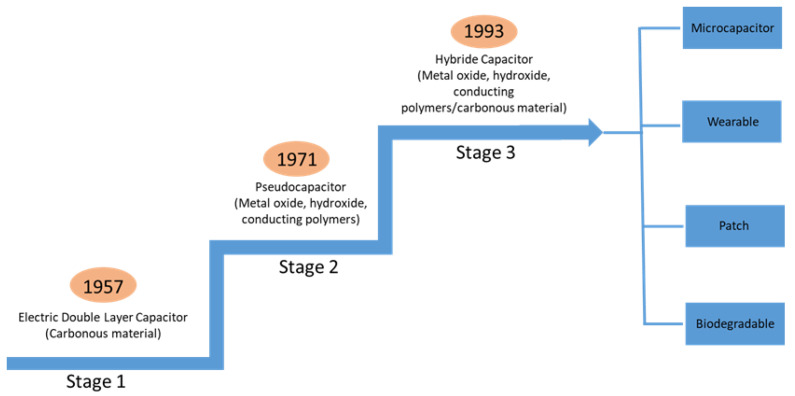
Development of supercapacitors and electrode materials. Reproduced with minor modifications from Ref. [2] with permission from MDPI AG.

The capacitance of supercapacitors can reach thousands of Farads (F). Unlike batteries, charge storage in supercapacitors is controlled by surface reaction instead of ion diffusion in the material. Therefore, supercapacitors are better than batteries in terms of power density in the same volume. Supercapacitors can be used in smart applications such as wearable devices, sensors, and portable energy storage systems. A higher specific surface area in supercapacitors can provide not only higher capacitance but also higher energy density [1]. While the capacitance of conventional capacitors is in the range of micro- and milli-Farads (μF and mF), in supercapacitors it can reach thousands of Farads. Figure 1 shows the history of supercapacitor development and the revolution of active materials [2].

**Figure 2:**
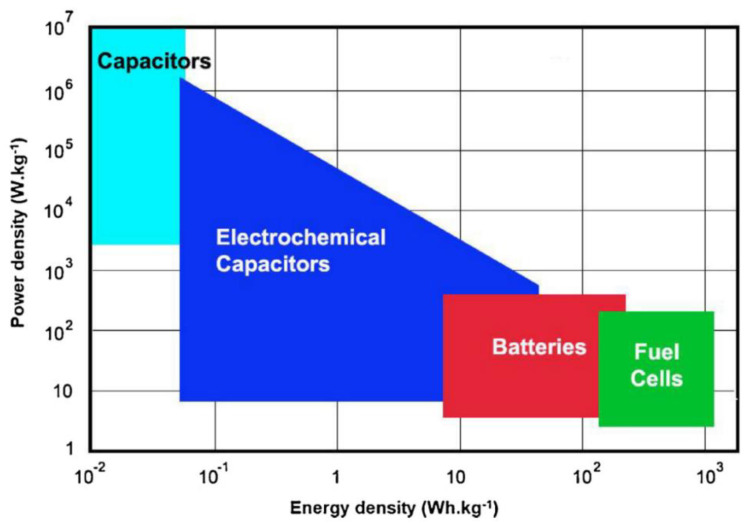
Ragone chart: Power density vs. energy density of energy storage devices. Reproduced from Ref. [7] with permission from Elsevier.

SCs provide an excellent balance between power density and energy density by bridging the gap between batteries that have high energy density and traditional capacitors that have high power density [3,4,5]. They can be used as an energy storage source in outlying places where there is no public energy network because wiring costs are too high, or in places where wiring is dangerous. They can also be used as a power supply in portable devices such as cellphones, notebooks, and electric or hybrid vehicles [6]. The Ragone chart in Figure 2 shows the difference between the level of energy density and power density of different energy storage devices [7].

An SC, also called an ultracapacitor, is an electrical component that can store electrical energy in its structure. SCs have been developed since the 1950s of the 20th century. The first SC had a capacity of around 1 F, which was patented in 1971 by Standard Oil of Ohio (SOHIO). The first commercial product was marketed by Panasonic in 1982 and was named Gold Cap [7]. The Gold Cap had high equivalent series resistance (ESR). In 1992, a commercial SC, called electrochemical double-layer capacitor, or EDLC at the time, with low ESR, was introduced to the market by Maxwell Laboratories, with the brand Boost Cap and a nominal capacity of about 1 kF [7]. Today’s SCs have capacities higher than several thousand F and are used in devices that need to release a huge amount of energy in a short time. They can also be used in electric and hybrid vehicles, fuel cell vehicles, electronic devices, energy harvesting systems, solar cells, and wind turbines [8].

The selection of electrode material and appropriate electrolytes, such as polyvinyl alcohol (PVA), which is one of the commonly used water-soluble polymeric electrolytes, should follow some criteria [9,10]. Compared with aqueous electrolytes, organic and ionic gel electrolytes can increase the working potential of supercapacitors because of their wide potential window [11,12]. The critical factors that have to be taken into consideration include operating potential, chemical stability, ionic conductivity, solubility, working temperature window, and viscosity, as well as mechanical properties [13].

**Figure 3:**
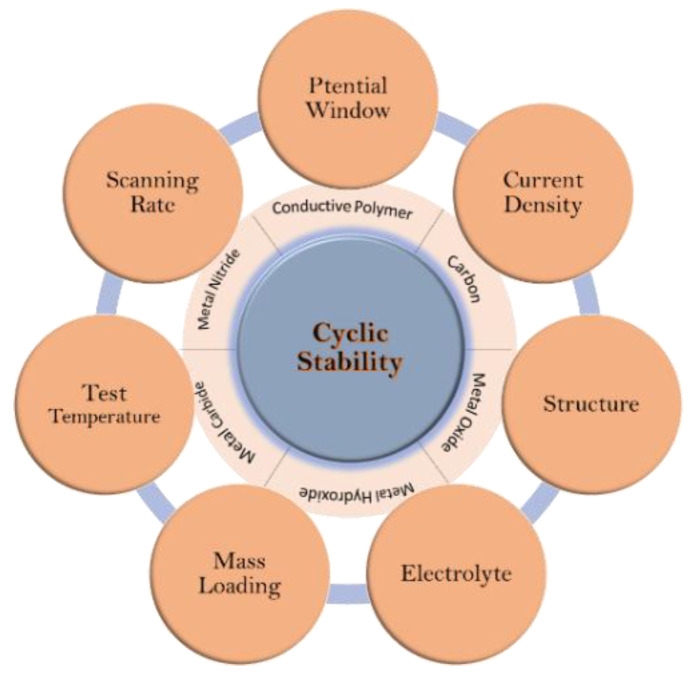
Factors affecting cyclic stability of supercapacitors. Reproduced from Ref. [14] with permission from the Royal Society of Chemistry.

Cycle stability is another factor that should be taken into consideration. This factor is related to the energy storage mechanism, which can be categorized in three different groups. The first group is the EDLC type, such as carbon materials, in which the charge/discharge process is based on physical absorption of ions at the interface of the electrode and electrolyte. Stability of these kinds of materials can decrease significantly during long cycles. The second group has battery-type and pseudocapacitive behaviors, such as metal oxides, metal carbides, and metal nitrides, which is based on self-activation reactions. During a self-activation process, specific capacitance increases with respect to the initial value, but drops to the initial value or less than 100% after finishing the self-activation step. The third group belongs to conductive polymers, of which the energy storage mechanism is based on reversible redox reactions, i.e., the doping–dedoping of p-type or n-type counter ions that produce Faradic current. But because of the volume expansion/contraction during charge/discharge cycles, capacity retention is less than 100% for this group of materials [14]. Figure 3 shows a brief classification of different factors that can affect cycle stability.

Conducting polymers with conjugated structures, such as polypyrrole (PPy), polyaniline (PANi), and polythiophene (PTh) and their derivatives, are interesting materials to be used as active materials in supercapacitors because of their unique electrochemical properties. They can also be composed with other electrode materials, such as graphene, metal oxides, and carbon nanotubes (CNT), to adjust their properties to achieve special functionalities [15,16,17].

Other than electrolytes and electrode materials, the structural design of an electrode also plays a key role in supercapacitor performance. For example, a 3D structure increases the surface area and can facilitate the charge and discharge process [18]. The substrate on which the electrode material is loaded also plays an important role. It can improve flexibility, stretchability, self-healing ability, and waterproof functions of supercapacitors [19,20,21].

To design a high-performance supercapacitor, it is critical to select the electrode material, electrolyte, and structure according to its application. Current research shows that the available supercapacitors are still not satisfactory in large-scale applications. There are important issues to be resolved, such as energy density, power density, durability, and good mechanical properties [13]. Study of the electrochemical process between electrolyte and electrode material, electrical double-layer configuration, charge–discharge, mass transfer mechanism, and all electrochemical reactions related to pseudocapacitance is of significance to establish the theoretical foundation of polymer-based supercapacitors [22,23,24].

Despite many achievements in this field, the research about supercapacitors, especially flexible supercapacitors, needs to be improved for large-scale industrial applications. Developing novel materials with high electrochemical capacitance, long durability, good mechanical properties, and fast charge/discharge ability is essential in the case of flexible supercapacitors. Based on updated information, this review provides the state of the art of conductive polymer-based electrode materials and supercapacitors, focusing on material chemistry, fundamentals governing conductive polymer conductivity, critical factors affecting capacitance, typical characterization methods, and how to extract data to calculate typical performance parameters describing electrodes and supercapacitors. It provides researchers in the field with updated information and furnishes beginners with a relatively comprehensive background and tools.

## 2. Supercapacitors Based on Conducting Polymers

Conducting polymers (CPs) are exciting candidates for supercapacitor electrodes. PPy, PANI, and PTh are the main CPs that have been heavily investigated in recent years [25,26].

A supercapacitor consists of two electrodes, a separator, and electrolyte. The charge can be stored at the interface of electrode and electrolyte in the form of an electrical double layer, and its capacitance can be calculated from Equation (1):

(1) $$C=\frac{A\varepsilon }{d}$$

where:

C: capacitance between two electrodes;
A: area of the two surfaces of the electrodes that face each other;
ε: dielectric constant of the electrolyte; and
d: separation distance between two electrodes.

Energy density, power density, and cycle life are the critical parameters to evaluate the performance of a supercapacitor, which can be calculated from the below equations. Based on Equation (2), two different types of capacitance in their structure (C_p_ and C_n_) are connected in series and make the total capacitance (C_T_) [27].

Total capacitance:

(2) $$\frac{1}{C_{T}}=\frac{1}{C_{p}}+\frac{1}{C_{n}}$$

where:

C_p_: positive electrode/electrolyte; and
C_n_: negative electrode/electrolyte.

Actually, capacitance shows the amount of charge in the presence of electrical potential, which depends on the amount and the surface area of the active material. Capacitance can be calculated based on weight (C_m_, F/g), area (C_s_, F/cm^2^), or volume (C_v_, F/cm^3^) [22], as shown in Equations (3)–(5).

(3) $$C_{v}=\frac{1}{\vartheta r∆V}\int_{V_{0}}^{V_{0}+∆V}IdV\;=\frac{I∆t}{\vartheta ∆V}$$

(4) $$C_{m}=\frac{1}{mr∆V}\int_{V_{0}}^{V_{0}+∆V}IdV\;=\frac{I∆t}{m∆V}$$

(5) $$C_{s}=\frac{1}{sr∆V}\int_{V_{0}}^{V_{0}+∆V}IdV=\frac{I∆t}{s∆V}$$

where r: scan rate (V/s);

V_0_: lower potential limit (V);
ΔV: potential window (V);
I: current (A); and
Δt: discharge time (s).

Current and test duration must be normalized by weight, volume, or area.

Other parameters that are very important in supercapacitors are output power and stored energy, which are shown in the Equations (6) and (7) [22]:

(6) $$E_{X}=\frac{1}{2}C_{X}{∆V}^{2}$$

(7) $$P_{X}=\frac{1}{4R}{∆V}^{2}=\frac{E_{X}}{∆t}\;$$

X = m for mass, s for area, or v for volume

### 2.1. Electric Double-Layer Capacitors or EDLC Supercapacitors

**Figure 4:**
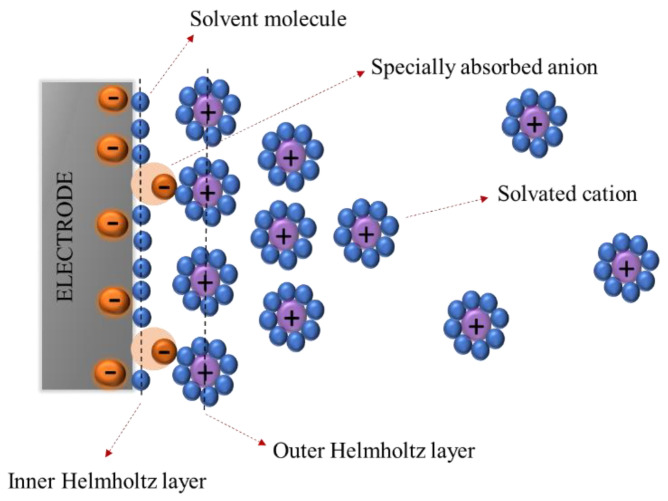
Helmholtz double layer in ELDC supercapacitors.

EDLCs are the most common supercapacitors in the marketplace. The charge transfer mechanism is based on electrostatic interactions. In these types of supercapacitors, a layer called a Helmholtz double layer is formed at the interface between electrode and electrolyte, where energy (charge) is electrostatically stored, as illustrated in Figure 4. Because there is no electron exchange and redox reaction between electrode and electrolyte, the current discharged by EDLCs is non-faradaic. The critical parameters that can affect EDCL capacitance are electrode surface area and Helmholtz layer thickness. The most used electrode material is activated carbon (AC) because of its large surface area and low price. The dielectrics used in EDCLs can be liquid electrolytes, and there is not any electrochemical reaction on the electrode during the charging/discharging process [6].

### 2.2. Pseudo-Supercapacitors or Faradaic Supercapacitors

In these types of supercapacitors, a reversible faradaic process of redox reaction, or reversible electrochemical doping–dedoping, and reversible adsorption occur on the electrode surface. Reversible adsorption of hydrogen can occur on the surface of a gold or platinum electrode. A reversible redox reaction can occur on the surface of metal oxide electrodes, such as RuO_2_, MnO_2_, CoO_3_, NiO, and Fe_3_O_4_. And reversible electrochemical doping–dedoping can occur on the surface of conductive polymer electrodes, such as PPy, PANi, and PTh. All of these processes generate faradaic current [27,28,29].

With the electrochemical or faradaic process, working voltage is extended and specific capacitance is increased [30]. Conway et al. have shown that a faradaic supercapacitor can have a capacitance of 10 - 100 times higher than electrostatic supercapacitance [31].

As mentioned before, in these types of supercapacitors, the charging/discharging process occurs on the electrode surface in the form of both reversible redox reactions and an electric double layer. Because of the doping and dedoping process during redox reactions, the electrode is stressed and degrades faster than electrostatic supercapacitors. The difference in charge/discharge mechanisms between electrostatic supercapacitance and pseudocapacitance is shown in Equations (8) and (9). Actually, in an electrostatic capacitor (Equation (8)) [23], there is no charge transfer and ion exchange across the electrode and electrolyte interface, while in pseudocapacitors (Equation (9)), a reversible chemical reaction occurs at the electrode surface.

(8) $$E_{p}+E_{n}+A^{-}+C^{+}\overset{\mathrm{Charging}}{\underset{\mathrm{Discharging}}{⇌}}E_{P}//A^{-}+E_{n}//C^{+}$$

(9) $${\left\{{\left[X\right]}_{n}^{0}\right\}}_{m}+A^{-}\overset{\mathrm{Charging}\left(-e^{-}\right)}{\underset{\mathrm{Discharging}\left(+e^{-}\right)}{⇌}}{\left\{{\left[X\right]}_{n}^{+}A^{-}\right\}}_{m}$$

### 2.3. Hybrid Supercapacitors

By combining pseudocapacitance and electrostatic capacitance, another type of supercapacitor can be created, which are called hybrid supercapacitors. With this combination, one can increase both volumetric and gravimetric energy densities. Consequently, a higher amount of current can be produced. Additionally, due to the faradaic reaction from the pseudocapacitance part, energy density is also increased. Right now, hybrid supercapacitors still need to be improved and are not commercially available [32]. Table 1 compares the characteristics of these three types of supercapacitors and lithium-ion batteries.

## 3. Electrode Material

To design a high-performance supercapacitor, choosing the right active material is very important [35]. Selection of polymers, polymer chain modification, and polymer blending with other materials are important techniques that are essential to create new polymer structures or compositions based on their final usage. These modifications can improve mechanical, electrical, and electrochemical properties [36].

The electrode materials chosen for supercapacitors must have critical characteristics, such as high conductivity, high electrical capacity, good mechanical property, high corrosion resistivity, good chemical stability, being environmentally friendly, and low cost. Among the various materials, conducting polymers have attracted significant attention because of their unique and fascinating characteristics.

### 3.1. Conducting Polymers (CPs)

CPs are organic materials with a conjugated band system that can conduct electricity without any conductive filler. The most common CPs include PPy, PANi, and PTh. CPs can be synthesized through chemical polymerization in the presence of oxidant and dopant or through electrochemical polymerization in the presence of dopants and monomers in a specific solvent [37]. To increase the conductivity and electrochemical performance of CPs, they have to be doped with appropriate dopants [38]. The doped CPs can be n-type or p-type, and the charge/discharge process occurs simultaneously with the doping and de-doping processes. Equations (10) and (11) show the charging process in the n-doping and p-doping states, respectively [38]. The discharge process is the reverse of these equations.

(10) $$C_{p\;}\;\to C_{p}^{n+}(A^{-}{)}_{n}+ne^{-}\;(p\;doping)\;$$

(11) $$C_{p}+ne^{-}\;\to \;(C^{+}{)}_{n}C_{p}^{n-}\;(n\;doping)$$

The polymer’s nature determines the type of dopant. PPy and PANi cannot be n-doped because of inappropriate potential, and they can only be p-doped. However, PTh is p- and n-dopable. Generally, the specific capacitance of n-doped PTh is lower than that of the p-doped form. In low potential (less than −2.0 V vs. Ag/AgCl), PTh is n-doped. However, n-doped PTh is unstable and can’t be used in practice, even its conductivity is comparable with that of the p-doped form [38]. Therefore, choosing the right material for a specific application is very important [38].

The conductivity of these polymers can be tuned from 10^−10^ up to10^4^ S/cm with different dopants or doping levels [39,40,41]. Based on their electrical conductivity and redox states, they can be used as the active materials of pseudocapacitors. They can have a large specific capacitance, such as 1284 F/g for PANI [42], 480 F/g for PPy [43], and 210 F/g, for PEDOT [44]. Table 2 shows a list of typical conducting polymers with their chemical structures [45].

#### 3.1.1. Polypyrrole

Among CPs, PPy is very attractive because of its balanced properties, such as thermal stability, high conductivity, environmental stability, and ease of synthesis [40]. PPy is readily synthesized on various substrates of various shapes to provide a high electrochemical performance [46]. The charge storage capability of PPy can be increased by improving the ion diffusion rate and contact surface area [47]. Based on these unique advantages, PPy has been considered a promising candidate to fabricate flexible and light-weight supercapacitors. However, the poor cyclic stability, low electrical conductivity of PPy composites, and the poor mechanical properties of pure PPy are still significant challenges [48].

To overcome such problems, many different techniques have been designed, for example, PPy with designed morphology or PPy composed with carbon materials or metal oxides. The electrical capacitance and cyclic stability of PPy must be improved before it can be used in energy storage devices.

**Figure 5:**
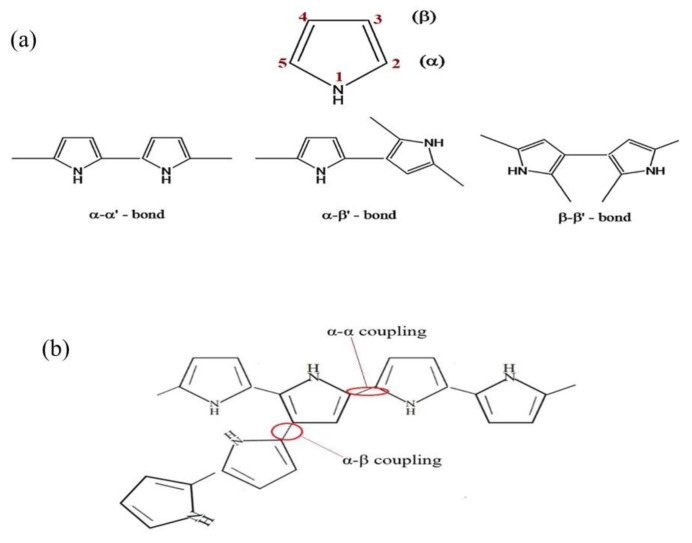
(**a**) Pyrrole and the possible structures of pyrrole dimers, (**b**) α-α and α-β couplings leading to chain branching and crosslinking in PPy structure. Figure 5a is reproduced from Ref. [53] with permission from Elsevier.

Polymerization conditions, such as the type of polymerization, temperature, potential range, oxidant, solvent, etc., are very important. The ideal polymerization of pyrrole occurs when the α carbons (Figure 5) are oxidized, either by chemical oxidants or through an oxidative electrical potential, into cation radicals. These cation radicals then join one with the other and deprotonate to form a dimer. The dimers will be oxidized again to form cation radicals and join to form oligomers [49], and so on. However, PPy can have a high degree of crosslinking [50,51,52]. As shown in Figure 5, there are two reaction sites, α (positions 2 and 5) and β (positions 3 and 4), on a pyrrole ring. Chain propagation at α sites leads to a regular and linear structure, while propagation involving β sites leads to irregular structures and crosslinks. Since high temperatures can increase the probability of the reactions at the β position, if a highly ordered structure of PPy is desired, a low reaction temperature is preferred to reduce the degree of crosslinking [53].

**Figure 6:**
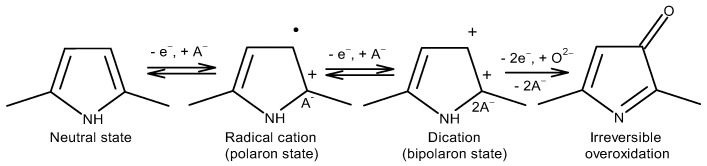
Oxidation states of PPy.

PPy has different oxidation states. As shown in Figure 6, it can switch reversibly between the first three states during the redox process. There is no change in covalent bond in the first three states. The last state, which is called the overoxidized state, is irreversible. The irreversible state occurs because one or more oxygen atoms are covalently attached to the pyrrole ring, leading to the loss of the structural conjugation and hence the decrease in conductivity. In fact, undoped PPy can easily react with atmospheric oxygen and become overoxidized.

Polymers are insulators in general. However, in the case of conducting polymers, when they are doped with the proper counterions, they become electrically conductive [54,55]. When doped, PPy obtains a dynamic electrical structure that can transport electricity from one side to another with the help of polarons or bipolarons [56].

Next, the critical parameters that affect the final conductivity of the CPs will be discussed in detail. These parameters include the experimental conditions, such as the polymerization method, polymerization temperature, electrode material in case of electrochemical polymerization, solvent, counterion/electrolyte, monomer substitutions, oxidant type, nature of the counterions, molar ratio of dopant/monomer, and application temperature of the CPs [57].

Dian and Lacroix have shown that during polymerization, nucleophiles can decrease conductivity and mechanical properties. Water molecules can act as nucleophiles and attack the pyrrole ring to form carboxyl groups, breaking up the conjugation of polymer chains [58].

**Figure 7:**
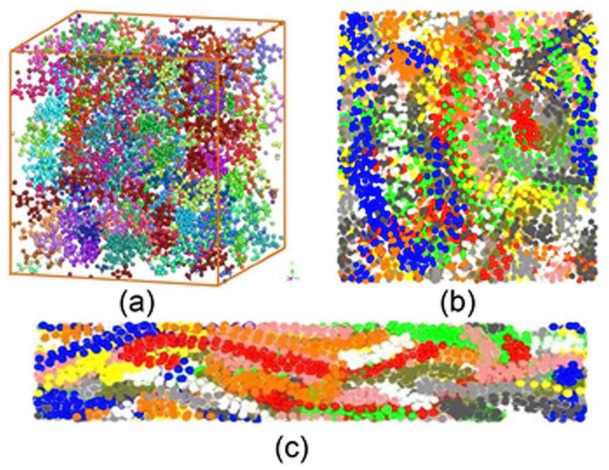
Stretch effect on polymeric chain, showing: (**a**) 3D unit cell of the polymer in unstretched state, (**b**) projection of the unstretched cell to XY plane, (**c**) stretched sample. Colors represent different polymer chains. Reproduced from Ref. [62] with the permission of American Physical Society.

Counterions also have a dramatic effect on conductivity [59]. The presence of bulky functional groups on the pyrrole ring can decrease the degree of conjugation and, consequently, conductivity. To avoid such negative effects, it is needed that these groups be separated at least by four carbons in the polymeric chain, and it is better to use a flexible chain as a linker between the pyrrole ring and the functional group [60]. Based on some studies, thermal conductivity can be increased by stretching the polymer because of polymer chain alignment [61]. Stretching makes polymer chains aligned in the stretch direction. As a result, thermal transfer becomes more efficient along the oriented polymer chains (Figure 7) [62].

Synthesis temperature affects the conductivity of PPy. By reducing the temperature, the conductivity of PPy increases. This increment is because of reducing the number of side reactions leading to overoxidation and non-planar structures [61,63].

Doping level directly affects conductivity. By increasing the doping level, conductivity can increase as high as 15 orders of magnitude [64]. A dopant can enter the PPy structure during or after polymerization to create a conjugation defect along the polymeric backbone. This defect is involved in the double-bond rearrangement of the conjugated system by forming a polaron or a bipolaron, which is the charge carrier that transfers the charge along the chain [65,66]. The conductivity normally increases with an increase in doping level that is affected by the dopant/monomer molar ratio and the nature of the dopant. Dopant can be either added into polymerization solution or generated by the oxidant. For example, by using ferric chloride (FeCl_3_) as the oxidant, it forms a donor–acceptor complex in the conjugated system and leaves a chlorine anion as a counterion.

The polarons act as charge carriers and are formed at a low concentration of the dopant. The mobility of the polarons is not high, and consequently, the conductivity of the PPy is relatively low as well. By increasing the amount of dopant, more Cl^−^ anions are inserted into pyrrole rings to create more polarons. When the polymer chain becomes crowded with polarons, they form bipolarons that have a higher energy level. This process increases the number of charge carriers and also their mobility, leading to an increase in conductivity [67].

Application temperature also affects conductivity. It has been shown that a high-temperature application environment can increase the conductivity of conductive polymers. It was found that at low temperatures, charge carriers are electrostatically bound to the counterions, such as Cl^−^ anions, and therefore, they are not expected to move fast. However, at higher temperatures, the charge carriers have enough energy to overcome the electrostatic attachment to the counterions, and consequently, they can easily move, leading to higher conductivity [68]. Electron spin resonance (ESR) studies show that by increasing the temperature in doped PPy, the g-value of the polymer increases linearly from 1.9504 to 1.9925 in a temperature range of 30–200 °C [69]. The g-value is a factor that has direct relation to the specific energy of a particle; this value in a free electron is 2.0023, and the g-values of free radicals are very close to this value [69].

Polarons and bipolarons are a kind of self-localized defection associated with their quantum state in an energy gap. The quantum state and energy gap of these two particles are different in terms of spin and charge. A polaron has a spin of ±1/2 and an electric charge of ±e; however, a bipolaron is spinless and its electric charge is ±2e [70,71].

**Figure 8:**
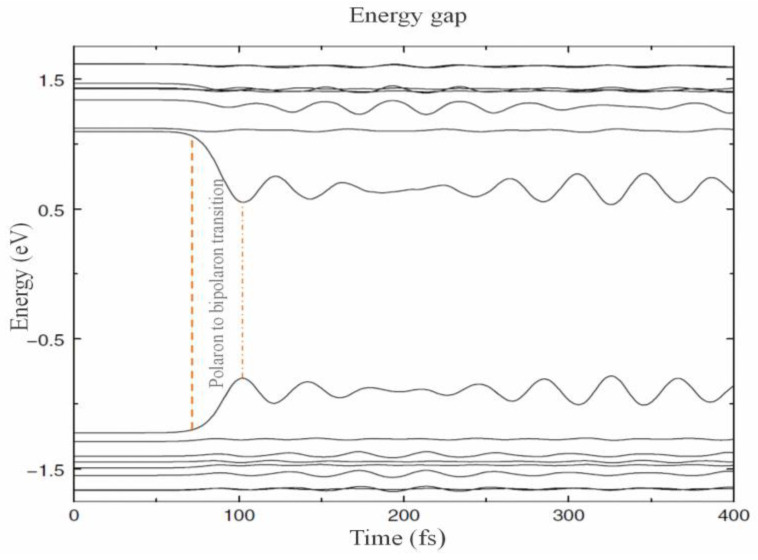
Time evolution of the energy level inside and around gap relating to an adiabatic transition of polaron levels (t < 80 fs) into bipolaron levels (t > 100 fs). Reproduced from [72] with permission from Springer Nature.

Transition from the polaron level (t < 80 fs) to the bipolaron level (t > 100 fs) occurs after the adiabatic removing of an electron. Figure 8 shows the time evolution of this transition by decreasing energy levels and energy gap. The small oscillation of the energy levels is because of lattice oscillation by hole perturbation [70,72].

**Figure 9:**
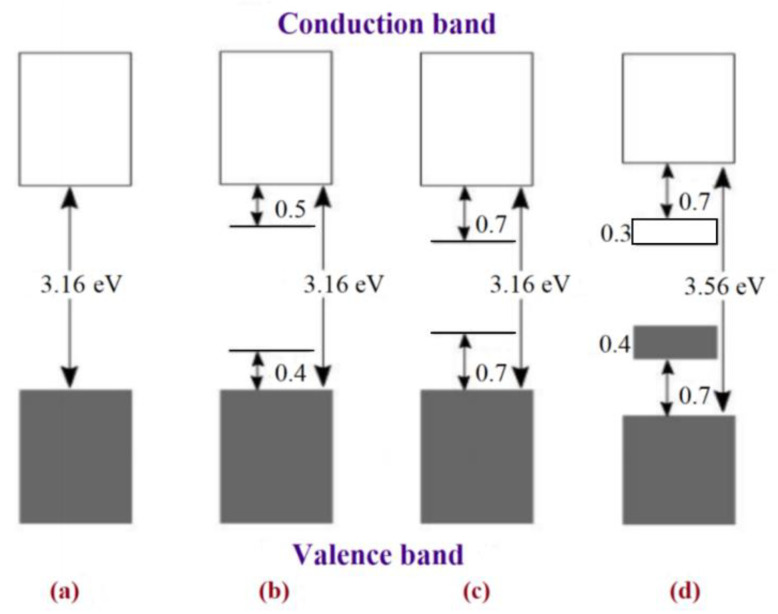
Electronic bands illustrating (**a**) undoped; (**b**) polaron; (**c**) bipolaron; and (**d**) fully doped states of PPy. Reproduced from Ref. [74], CC-BY 3.0.

The separation of energy bands determines what is metal, semiconductor, or insulator, as illustrated in Figure 9. In metals, the band gap between the valance band (VB) and conducting band (CB) is zero (Eg = 0 Ev); that is why electrons can move freely in metals. However, in insulators, this gap is larger than 3.16 eV, so large that the electrons do not have enough energy to jump from VB to CB. In semiconductors, the gap between VB and CB is decreased by a treatment known as doping that generates electrons and holes as charge carriers in inorganic semiconductors and polarons and bipolarons as charge carriers in CPs [73,74,75].

#### 3.1.2. Polyaniline

**Figure 10:**
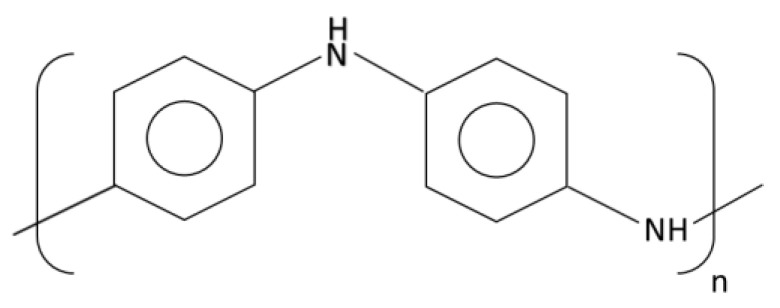
Polyaniline chemical structure.

Polyaniline, or PANi, also called aniline black, is another CP that has been used in energy storage applications. It can be synthesized by chemical and electrochemical polymerization methods. Its light weight, high conductivity, low cost, and good mechanical properties make PANi an interesting material to be used in supercapacitors. Figure 10 shows the chemical structure of PANi.

PANi can be deposited on the surface of metal oxides (MOs)/carbon to form a PANi/MOs/nanocarbon ternary hybrid [76]. Because of the color change of PANi during the redox process, it can be used in electrochromic supercapacitors. Comparing the three popular CPs, PPy and PTh are more stable than PANi and can be synthesized directly in doped form [27]. According to the studies on the PANi synthesized under different conditions, only the one that has the potential window of 0.8–1.0 V can be used in supercapacitors, and any PANi with a potential window of less than 0.6 V is not in the supercapacitor class. This is because that energy density is proportional to the square of the cell voltage (E = (CV^2^)/2), meaning that a low potential does not provide enough energy to a supercapacitor [77,78]. PANi can be doped in n-type or p-type. Doping can be done during polymerization or after polymerization [79]. Some dopants in PANi can attach covalently to the polymeric chain and cannot be removed during the subsequent electrochemical redox process, which blocks a portion of the capacitance of PANi. Dedoping can work in favor of electroactivity because it can form structural micropores, consequently increasing capacitance. On the other hand, initial doping can affect the morphology of CPs, so that directly affects the overall performance of the supercapacitor [80,81,82,83,84].

Morphology is one of the crucial factors that can affect the electrochemical behaviors of PANi. Since the electrode–electrolyte interface area plays a significant role [85], by increasing the surface porosity of the PANi electrode, the specific capacitance of the supercapacitor can be increased. For example, Sharma et al. synthesized a nanoporous PANi with a specific capacitance of 410 F/g [86]. Another method by which we can increase the surface area and then the specific capacitance is to deposit PANi on a substrate of high surface area, such as a highly ordered metal oxide, porous carbon, or a template, such as a nanofiber template that can be removed after polymerization [87,88,89,90].

Nanostructured PANi can be easily synthesized by chemical and electrochemical methods. PANi has excellent electrochemical cyclability and reversibility, which make it appropriate to be used in a supercapacitor [79]. There are many parameters that can affect the electrochemical properties of PANi during both chemical and electrochemical polymerizations, such as polymer chain length, crosslink degree, types of dopant, doping level, morphology, and diffusion pathways. These characteristics can be controlled with different parameters, such as temperature, concentration of the solution, and dopant [91]. It was reported that a crosslinked PANi electrode retained 100% of its specific capacitance after 1000 cycles [92].

There are generally three main types of PANi nanocomposites, i.e., carbon/PANi, metal oxide/PANi, and metal oxide/carbon/PANi, which have been investigated for use in pseudocapacitors.

In the case of carbon/PANi composite, carbon nanomaterials provide a high specific area, and PANi provides good electrical conductivity. PANi can be composited with carbon nanotubes, either in the form of single-walled (SWCNT) or multi-walled (MWCNT), to benefit from both the high surface area and high conductivity of the nanotubes [93,94,95]. A disordered polymeric chain can limit diffusion of electroactive species and redox site accessibility. In order to solve these kinds of problems, it is better to use ordered mesoporous carbon as a template to synthesize PANi on the external surface of ordered mesoporous carbon, which can lead to a high surface area structure and consequently, high capacitance [96,97]. The presence of appropriate functional groups at the surface of carbon nanotubes can form active sites for the growth and attachment of PANi, which is in favor of higher capacitance. Graphene with a 2D structure is an important type of carbon nanomaterial that has been widely investigated in PANi/graphene nanocomposites [98]. The nature of interactions between PANi and graphene affects the electrical capacity of the composite. When this interaction is non-covalent, such as Π- Π interaction (PANi and graphene have rich Π system), the cyclability of the supercapacitor drops by up to 45% after 1000 cycles [99]. PANi molecules can be deposited on the graphene sheet vertically with ordered alignment, leading to 1665 F/g specific capacitance [100].

Metal oxides are also good candidates for supercapacitors because they show pseudocapacitance behavior in a wide potential range. Metal oxides such as RuO_2_, MnO_2_, V_2_O_5_, Fe_2_O_3_, NiO, MoO_3_, and WO_3_ can be used in PANi-based supercapacitors [79]. In another approach, metal oxides can be used as oxidizing agents to initialize the polymerization. With this method, PANi has been shown to grow with the metal oxide and form a nanocomposite [101]. In such composites, PANi acts as a conductive material that compensates for the low electrical conductivity of metal oxides. The best example is MnO_2_, which has been used in batteries and supercapacitors and suffers from low conductivity. PANi effectively improved the electrochemical performance of the PANi/MnO_2_ nanocomposite [102,103].

There are also ternary composites made of PANi, metal oxide, and carbon, of which, all the advantages of the components, such as high conductivity, high specific surface area and high accessible electrochemical sites, are combined to achieve a high performance [104,105]. In these composites, metal oxides are attached to the graphene surface to form a good substrate, on which PANi can grow vertically. A research group has shown a specific capacitance of 1360 F/g with a graphene/ZrO_2_/PANi system [106,107].

#### 3.1.3. Polythiophene (PTh)

**Figure 11:**
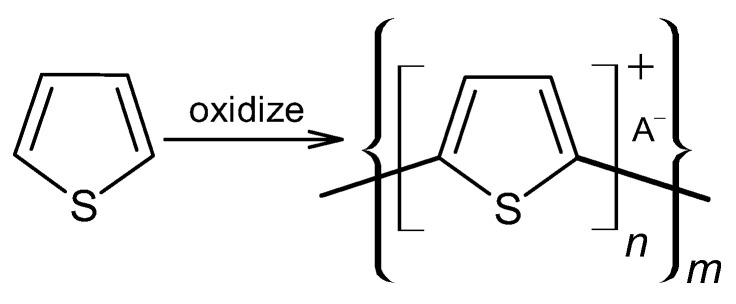
Polythiophene-forming process.

PTh is a conducting polymer with high environmental stability and tunable conductivity. PTh can be synthesized in the form of powder or film [108]. There are many different methods to synthesize PTh, including chemical and electrochemical [109], photochemical [110], ultrasonic-assisted, and template-assisted syntheses [110,111]. PTh is synthesized from thiophenes, as showed by Figure 11.

**Figure 12:**
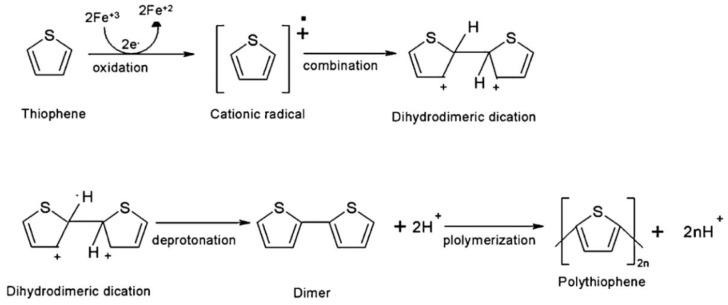
Mechanism of polythiophene polymerization. Reproduced from [117] with permission from John Wiley & Sons.

Normally, n is between 2 and 4, A^−^ is a counterion required to maintain the oxidation state, and m is the number of repeat units and is related to molecular weight. The electrochemical polymerization of PTh is a process considered a paradox, meaning that at the potential that thiophene needs to be oxidized, the synthesized polymeric chain can be overoxidized, changing the final chemical and physical properties. At a constant current or potential, the synthesized material is a mixture of the oxidized and overoxidized polymers. Some research groups have been able to reduce the overoxidation by using bithiophene or terthiophene instead of thiophene. However, the results have shown that the PTh that is synthesized with these initial dimers or trimers has lower conductivity [112,113,114]. Fu et al. synthesized a PTh film with the specific capacitance of 110 F/g by the electrochemical polymerization method [115]. Senthil Kumar et al. reported a PTh film with a specific capacitance of 70 F/g by the chemical polymerization method [116]. Figure 12 shows the reaction mechanism of PTh formation in chemical polymerization [117].

The specific capacitance of the electrode made of PTh derivatives (ex. PEDOT) is relatively lower than that made of PANi and PPy [38]. PTh can also be n-doped or p-doped. Consequently, they can be used as the negative electrode material or the positive electrode material. Comparing n-doped and p-doped materials, the n-doped PTh has lower capacitance and conductivity than the p-doped ones [38]. The n-doped PTh is produced at low potential and is sensitive to oxygen and water. Consequently, it can be easily oxidized and can severely self-discharge, which degrades the electrochemical performance of the electrode [118,119]. In order to overcome this problem, an electron-withdrawing group can be added to the thiophene ring so that the n-doped PTh can be synthesized at less negative potential [120].

Among the three CPs mentioned above, PPy often shows a high capacitance and cyclic stability in the literature, while PANi may show a high conductivity and PEDOT a high chemical stability. For example, flexible electrodes made of PPy/FeCl_3_ nanorods coated on cotton fabrics recorded 578 F/g at 0.2 A/g [121]; PEDOT deposited on a carbon paper showed 126 F/g at 1mA/cm^2^ [122]; and nanostructured PANi deposited on graphite electrodes demonstrated 460 F/g at zero scan rate (by interplotting) [123]. However, the values in the literature often cannot be directly compared because of variations in other parameters, such as current density, scanning rate, area vs. weight, frequency, etc.

## 4. Electrode Arrangement: Two vs. Three Electrodes

Electrode arrangement plays a crucial role in the characterization of electrochemical devices. The choice between two-electrode or three-electrode systems depends on the purpose of the measurement.

### 4.1. Three-Electrode System

Three-electrode systems are commonly used in the research and development of electrode materials. The material of interest is used as the working electrode, together with a reference electrode, such as silver chloride (Ag/AgCl), placed near the working electrode, and a counter- (also called an auxiliary) electrode that should have a sufficient surface area, such as a platinum mesh. A potentiostat ensures that most current passes through the working and counter electrodes, while the working potential is measured between the reference and working electrodes. This means that only a half cell (working electrode) is measured. This configuration can also accurately control the potential between reference and working electrodes. A three-electrode setup is often preferred during the initial testing and development stages of flexible electrodes [124]. This is because the precise control and accurate measurement of electrical potential provided by the three-electrode system are critical in understanding the electrochemical behaviors of the flexible materials [125]. Issues such as mechanical deformation, which can alter the electrochemical response, are more easily identified and mitigated using a three-electrode configuration [126].

### 4.2. Two-Electrode System

In a two-electrode system, there are only working and counter electrodes. The current passes between the two, and the voltage drop is measured. One can combine the reference lead with the counter-electrode lead to convert a three-electrode system into a two-electrode system. The main advantage of a two-electrode system is its simplicity and ease of implementation. Obviously, what is studied is the full cell instead of a single electrode. That is why this configuration is often used to study the performance of devices such as supercapacitors and batteries. It is particularly useful for large-scale energy storage devices where the primary focus is on overall performance rather than detailed mechanistic studies. However, this simplicity comes with certain drawbacks. The two-electrode setup does not allow for independent control or monitoring of the potential at the working electrode, which can lead to inaccurate measurements due to the potential drop across the counter-electrode. This can particularly affect the performance evaluation of flexible electrodes, where uniform current distribution and minimized resistance are crucial [127]. Flexible electrodes are typically implemented in a two-electrode configuration for wearable and portable devices due to their simplicity and low profile [128].

In conclusion, three-electrode systems offer precise control and measurement of electrochemical activities and are preferred in material research, and two-electrode systems measure entire electrochemical cells and are used to characterize full cell devices.

## 5. Electrolyte

**Figure 13:**
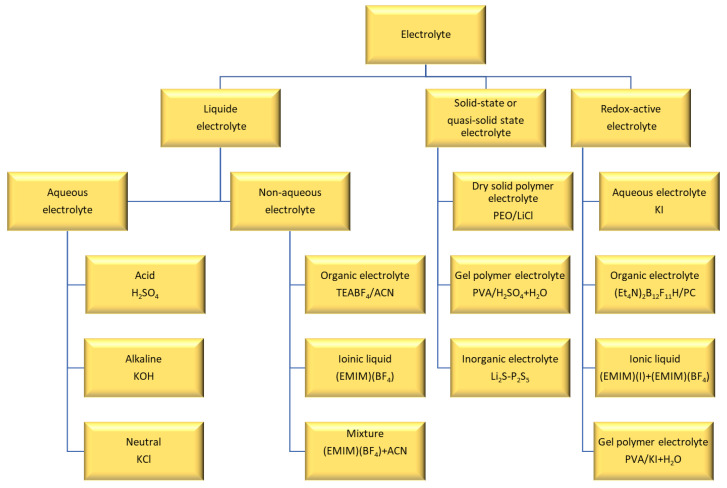
Classification of electrolytes for electrochemical supercapacitors. Reproduced from [13] with permission from Royal Society of Chemistry.

The electrolyte contains salts and solvents and plays a key role in supercapacitor performance. Important parameters are ion type, size, and concentration; solvent type; interactions between solvent and ions; interactions between electrolyte and electrode; and electrolyte potential window. The electrolyte can affect power and energy density and self-discharge of supercapacitors. Figure 13 below shows the electrolyte classification. Each electrolyte has some advantages and disadvantages. For example, aqueous electrolytes have high conductivity and high capacitance, but their working voltage is limited because of the relatively low decomposition potential of water (1.23 V) [129]. On the other hand, an organic electrolyte can be used at a higher potential, but they suffer from lower ionic conductivity. Solid-state electrolytes don’t have leakage problem, but sometimes they suffer from low ionic conductivity.

### 5.1. Liquid Electrolyte

Liquid electrolyte is classified into two main groups, i.e., aqueous electrolyte and non-aqueous electrolyte.

Aqueous electrolytes consist of acidic, alkaline, and neutral water solutions. Because they have narrow potential windows (1.0–1.3 V) [129], they are generally not used in commercial products but rather, in the research area, because they are inexpensive and easy to use without any special conditions. The important parameters that affect solvent conductivity and specific capacitance include ion (cation and anion) size in bare and hydrated states, and ion mobility. The potential window and corrosion degree of the electrolyte also have to be considered when choosing an appropriate electrolyte for a system. Table 3 shows a list of aqueous electrolytes, together with their performance in supercapacitors. The most frequently used acidic, alkaline, and neutral aqueous electrolytes are made of H_2_SO_4_, KOH, and Na_2_SO_4_, respectively. The operation voltage of these electrolytes is about 1 V. Parameters such as water decomposition voltage and water freezing and boiling points must be considered. One molar H_2_SO_4_ is the most common acidic solution reported in the literature because, in this concentration, it has the maximum ion conductivity, which can increase the specific capacitance of supercapacitors. EDLCs, in the presence of H_2_SO_4_, have higher specific capacitance than neutral aqueous electrolytes and organic electrolytes [130,131,132].

Generally, the specific capacitance and energy densities in EDLCs do not have a huge difference when using an alkaline (KOH) aqueous electrolyte or an acidic (H_2_SO_4_) electrolyte [132]. A KOH solution with a concentration of 6 M has high ionic conductivity and so has frequently been used among other alkaline solvents. Compared with alkaline and acidic solvents, neutral electrolytes have lower ionic conductivity, which causes lower specific capacitance, but on the other hand, they can provide a higher operating voltage [133,134]. Table 3 shows the performance of some aqueous electrolytes.

**Table 3:** Aqueous electrolyte-based supercapacitors and their performance.

Electrode Type	Electrolyte	Cell Voltage (V)	Specific Capacitance (F/g)	Energy Density (W h/kg)	Power Density (W/kg)	Temp. (°C)	Refs.
Mesoporous MnO_2_	0.65 M K_2_SO_4_	1	224.88 at 1 mV/s	24.1	70	RT	[135], 2012
Mesoporous MnO_2_	1 M Li_2_SO_4_	1	284.24 at 1 mV/s	28.8	70	RT	[135]
Mesoporous MnO_2_	1 M Na_2_SO_4_	1	278.8 at 1 mV/s	28.4	70	RT	[135]
MnO_2_ nano flowers	1 M LiOH	0.6	363 at 2 mV/s	-	-	-	[136], 2015
MnO_2_@carbon nanofibers composites	0.5 M Na_2_SO_4_	0.85	551 at 2 mV/s (75 1C)	-	-	0–75	[137], 2013
AC	0.5 M Na_2_SO_4_	1.6	135 at 0.2 A/g	10	-	-	[133], 2010
AC	4 M NaNO_3_-EG	2	22.3 at 2 mV/s	14–16	500	0–60	[138], 2014
AC	1 M NaNO_3_	1.6	116 at 2 mV/s	-	-	RT	[139], 2014
AC	Na_2_SO_4_/0.5 M	1.6	135 at 0.2 A/g	10	-	-	[133]
AC fibers	1 M H_2_SO_4_	0.9	280 at 0.5 A/g	-	-	RT	[140], 2014
PANi-grafted rGO/AC	1 M H_2_SO_4_	0.8	1045.51 at 0.2 A/g	8.3	60,000	-	[141], 2014
Graphene/mPANi	1 M H_2_SO_4_	0.7	749 at 0.5 A/g	11.3	106.7	-	[142], 2014
PPy thin films	0.5 M H_2_SO_4_	1	510 at 0.25 mA/cm^2^	133	758	-	[143], 2014
Pristine flexible PPy membrane	Solid PVA/H_2_SO_4_/EG	0.7	191.7 at 0.5 A/g	14.1	181.9	RT	[144], 2021

Abbreviations: Temp: temperature; RT: room temperature; AC: activated carbon; PANi: polyaniline; rGO: reduced graphene oxide; PPy: polypyrrole; mPANi: mesoporous PANi film on ultra-thin graphene nanosheets; EG: ethylene glycol.

Another group is organic electrolytes, which have a high operation potential window between 2.5 and 2.8 V. This high operation voltage can improve both energy density and power density. Also, because they are less corrosive, organic electrolytes provide the possibility of using cheaper material for current collectors. However, the supercapacitors using organic electrolytes have a higher price and lower specific capacitance because of lower ion conductivity, flammability, and toxicity. Also, a complicated process of purification and assembly is required to remove residual impurities that can cause electrolyte degradation and self-discharging [145]. Generally, an organic electrolyte consists of a conducting salt dissolved in an organic solvent. Like for aqueous electrolytes, the nature of organic electrolytes, such as ion size, ion–solvent interaction, conductivity, and viscosity, can affect supercapacitor performance. The most used organic electrolytes in the literature are propylene carbonate (PC), ethylene carbonate-dimethyl carbonate (EC-DMC), and ethylene carbonate-diethyl carbonate (EC-DEC) [146,147,148]. Table 4 shows different organic electrolyte-based supercapacitors and their performance.

Ionic liquid electrolytes are another type of liquid electrolyte, consisting of melted cations and anions. Ionic liquids have high thermal, chemical, and electrochemical stability; very low volatility; non-flammability; and highly tunable physical and chemical properties [11,149]. Because of these attractive properties, they have attracted much attention.

**Table 4:** Organic electrolyte-based supercapacitors and their performance.

Electrode Material	Electrolyte	Cell Voltage (V)	Specific Capacitance (F/g)	Power Density (W/Kg)	Energy Density (Wh/Kg)	Temp. (°C)	Refs.
Electrode materials in double layer supercapacitors							
AC	1.5 M SBPBF_4_/PC	3.5	122 at 0.1 A/g	-	52	RT	[150], 2014
AC	1.6 M TEAODFB/PC	2.5	21.4 at 1 A/g	~1000	28 (20 °C)	−40 to 60	[151], 2012
AC	0.7 M TEABF_4_/ADN	3.75	25 at 20 mV/s	-	28	RT	[152], 2012
AC	1 M TEABF_4_/HFIP	-	110 at 1 mV/s	-	-	-	[153], 2012
Microporous carbide derived carbon	1 M NaPF_6_/(EC-DMC-PC-EA 1:1:1:0.5)	3.4	120 at 1 mV/s	~90	~40	−40 to 60	[154], 2014
Highly porous interconnected carbon nanosheets	1 M TEABF_4_/ACN	2.7	B120-150 at 1 mV/s	25,000-27,000	25	-	[155], 2014
Heteroatom doped porous carbon flakes	M LiPF_6_/(EC-DEC 1:1)	3	126 at 1 A/g	2243	29	RT	[156], 2014
Carbon (provided by Batscap)	1 M SBPBF_4_/ACN	2.3	109	-	-	−30 to 60	[157], 2013
Graphene-CNT composite	1 M TEABF_4_/PC	3	110 at 1 A/g	400	34.3	-	[158], 2013
Microporous TiC-CDC	1 M TEMABF_4_/(PC-PS 95:5)	2.7	100 at 10 mV/s (60 1C)	~1000	~25–27	−40 to 60	[159], 2014
Electrode materials in pesedoucapacitors							
PANi/graphite	0.5 M LiClO_4_/PC	1	~420 at 50 mV/s	-	-	RT	[160], 2013
MoO_3_ nanosheets	1 M LiClO_4_/PC	-	540 at 0.1 mV/s	-	-	-	[147], 2010
Nanoporous Co_3_O_4_ -graphene composite	1 M LiPF_6_/(EC-DEC 1:1)	-	424.2 at 1 A/g	-	-	RT	[161], 2014
Heterostructured poly (3,6-dithien-2-yl-9H-carbazol-9-yl acetic acid)/TiO_2_ nanoparticles composite	0.5 M Bu_4_NBF_4_/ACN	1.2	462.88 at 2.5 mA/cm^2^	-	89.98	RT	[162], 2014

Abbreviations: Temp: temperature; RT, room temperature; CNT, carbon nanotube; HFIP, 1,1,1,3,3,3-hexafluoropropan-2-ol; AC, activated carbon; ADN, adiponitrile; TEAODFB, tetraethylammonium difluoro(oxalato)borate; TEMABF_4_: triethylmethylammonium tetrafluoroborate; PS, 1,3-propylene sulfite; TiC-CDC: titanium carbide-derived carbon; SBPBF_4_, spiro-(1,10)-bipyrrolidinium tetrafluoroborate; EC: ethylene carbonate; DEC: diethyl carbonate; DMC: dimethyl carbonate; EA, ethyl acetate; PANi, polyaniline.

### 5.2. Solid and Quasi-Solid Electrolytes

A major type of solid electrolyte is polymer-based electrolytes, further classified into three different groups, including solid polymer electrolytes (SPEs), gel polymer electrolytes (GPEs), and inorganic electrolytes.

The most important advantage of solid electrolytes is the simplified packaging process, because they are liquid-leakage-free and can be used as an electrolyte and an electrode separator at the same time. Examples of solid electrolytes are Li_3_PS_4_, Li_7_P_3_S_11_, Li_10_GeP_2_S_12_, and metal oxides like LiNbO_3_, and LiTaO_3_ [163].

SPE electrolytes are composed of polymers and salts without any solvent. The parameters that should be considered to develop a solid-state electrolyte are thermal, chemical, and electrochemical conductivity, mechanical stability, and ionic conductivity.

Due to liquid in GPEs, some studies call this type of electrolyte a quasi-solid-state electrolyte [164,165]. A GPE is composed of a polymeric matrix, such as polyvinyl alcohol (PVA) and polyacrylic acid (PAA), and a liquid electrolyte that can be an aqueous electrolyte or an organic solvent. PVA is a linear polymer that can be dissolved in an aqueous solution, such as an alkaline solution (ex. KOH), a strong acid (ex. H_2_SO_4_), or a neutral solution (ex. LiCl), to make a hydrogel electrolyte. PVA hydrogel electrolyte has many interesting properties, such as high hydrophilicity, film-making properties, and non-toxicity. In addition, it has a low cost, and the preparation process is really easy [166]. Poly(acrylate) and PAA have also been studied as polymer hosts of electrolytes, and the resulting polymer hydrogels showed increased proton conduction when the protons entered an aqueous medium from polymer side chains [167,168].

A research group has shown the order of specific capacitance of a RuO_2_ electrode in the presence of different electrolytes, including poly(2-acrylamido-2-methyl-1-propanesulfonic acid (PAMPS), potassium polyacrylate (PAAK), PAA, as the following [167,169]:

PAMPS/H_2_O > 1 M H_2_SO_4_ (aqueous electrolyte) > PAA/H_2_SO_4_ > PAAK/H_2_SO_4_ > PAMPS/H_2_SO_4_

As it is obvious that the PAMPS/H_2_O electrolyte has the best performance among the others. This is because of the sulfonate groups at the side chains that can provide the best proton accommodation and, as a result, the highest capacitance [169]

Another type of quasi-solid-state electrolyte is called organogel electrolytes, which use organic solvents to replace water in order to increase the working voltage of the cell. Different polymers, such as polyethylene oxide (PEO) [170], poly(methyl methacrylate) (PMMA) [171], polyvinylprrolidone (PVP), polyether ether ketone (PEEK) [172], and copolymers [173,174], can be used to host organic solvents, such as polycarbonates (PC), ethyl cellulose (EC), dimethyl carbonate (DMC), dimethyl sulfoxide (DMSO), and dimethylformamide (DMF). This system can increase the cell voltage up to 2.5–3.0 V, which is higher than aqueous electrolytes that have cell voltages up to 1.3 V [169]. However, organogel electrolytes suffer from relatively low ionic conductivity.

The next group of quasi-solid-state electrolytes is ionic liquids (ILs), which have a large working potential window of up to 4.0 V [169]. When ILs are used as electrolytes, the IL is incorporated into a polymeric host. The properties of the electrolyte depend on the interactions between the IL and the host polymer. The polymers that can be used with ILs are the same as those used in organogel electrolytes, as mentioned above.

The last group of solid-state electrolytes is inorganic electrolytes. Inorganic electrolytes are not bendable and flexible, but on the other hand, they have good mechanical and thermal stability. A research group has reported a glass–ceramic electrolyte that is used as both an electrolyte and a separator and has high Li-ion conductivity [175].

### 5.3. Redox-Active Electrolytes

In these types of electrolytes, a redox reaction can occur in the electrolyte and increase capacitance [176]. This kind of electrolyte is prepared by adding suitable redox additives to an electrolyte system to improve the performance of the supercapacitor system. Some examples of these redox additives are Na_2_MoO_4_, Ce_2_(SO_4_)_3_, and 1,4-dihydroxyanthraquinonedone [177].

Heteropoly acids, such as phosphotungstic acid (PWA), can be used as redox-active electrolytes, which can increase proton conductivity and provide multiple redox–electron transfers [178,179]. The main problem with this type of electrolyte is self-discharging. The reason for it is the migration of the electrolyte between electrodes. In order to inhibit this migration, an ion-exchange membrane (e.g., Nafion) can be used as a separator or to use a redox-active electrolyte that can be converted to an insoluble form during the charging–discharging process [180]. These types of electrolytes can increase specific capacitance and, consequently, energy and power densities. They can provide an electron source and increase the speed of the Faradic reaction in pseudocapacitance electrodes. On the other hand, they can decrease cycle stability [181,182]. Potassium iodide (KI) and hydroquinone (HQ) are other examples of redox-active electrolytes.

Non-aqueous redox-active electrolytes are another type of redox-active electrolyte that can increase cell working voltage and energy density. The last group is solid redox-active electrolytes, which have almost the same electron transfer mechanism as the liquid ones.

### 5.4. Self-Healing Electrolytes

While solid and quasi-solid electrolytes are preferred in wearable and flexible electronics because of their low risk of leaking, they face the risk of structural damage during repeated deformation. Self-healing electrolytes can, ideally, automatically repair small damages such as cracks. The so-called self-healing electrolytes are largely based on two mechanisms: the release of pre-stored reactive chemicals inside a material (electrolyte), trigged by damages such as a crack. The released chemicals will eventually fill and bond to the crack. The re-bonding of the racks can also come from the electrolyte itself without involving the pre-stored chemicals. In such an electrolyte, the bonding between material molecules is dynamic and reversible. When the force generated by deformation exceeds the ultimate strength of the electrolyte, the bonds among electrolyte molecules are broken. However, when deformation disappears, if the broken molecules can return to their proximity, the bonds automatically reform among the molecules. A self-healing electrolyte is a special case of self-healing materials, which has been nicely reviewed recently [183,184,185].

## 6. Characterization Methods

Electrical conduction in CPs is much more complicated than in classical semiconductors because the doping of CPs involves a redox reaction that changes the chemical and electronic structures of the polymers. Also, CPs have a one-dimensional lattice that makes them amorphous, and the charge carriers are not electrons and often change with the degree of doping and the type of dopant. The appropriate methods used to evaluate electrode materials include cyclic voltammetry (CV), electrochemical impedance spectroscopy (EIS), conductivity measurement (ex: the four-point probe method), scanning electron microscopy (SEM), X-ray photoelectron spectroscopy (XPS), and Fourier transform infrared spectroscopy (FTIR).

### 6.1. Electrochemical Analysis

**Figure 14:**
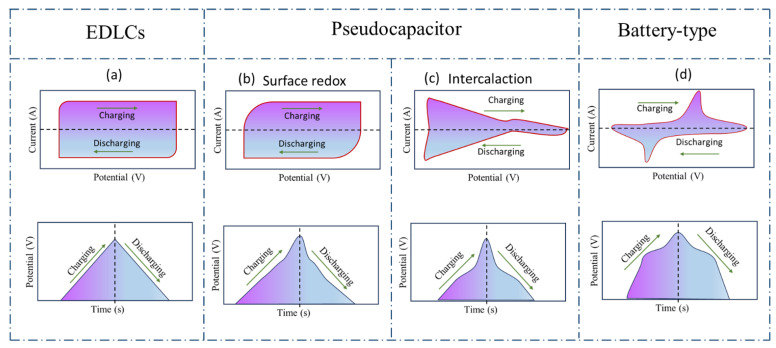
Comparison of electrochemical behaviors of different types of electrode materials based on CV cures (**up**) and GCD curves (**down**). (**a**) EDLCs, (**b**) and (**c**) pseudocapacitors, (**d**) battery-type. Reproduced from [14] with permission from Royal Society of Chemistry.

Based on electrode materials, their energy storage mechanisms can be different, which further affect cyclic voltammogram and charge/discharge curve shapes. As mentioned before, there are three categories of electrode materials. The first type is the ECDL type, in which a reversible adsorption/desorption process of electrolyte ions is formed at the interface between the electrode and electrolyte; no electrochemical reaction or phase change occurs so that their cyclic stability is generally good. The second type shows pseudocapacitance, in which charge storage is based on a reversible redox reaction on the surface of the material. Compared to the EDLC type, this type of electrode material has a larger specific capacitance, but its cyclic stability is relatively poor. In the third type, or battery-type materials, the electrochemical reaction is controlled by electrolyte ion diffusion that undergoes intercalation reactions of phase change ions. Battery-type materials show high energy density but poor cyclic stability [14]. The difference in electrochemical behaviors of electrode materials is shown in Figure 14.

#### 6.1.1. Electrochemical Impedance Spectroscopy (EIS)

**Figure 15:**
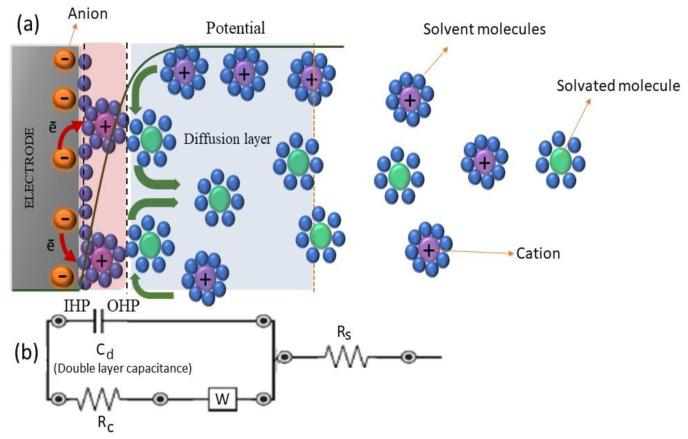
Components of a simple electrochemical system, showing: (**a**) electric and electrochemical phenomena occurred in the solution and interface area; (**b**) modeled circuit based on these phenomena. C_d_: double layer capacitor, R_C_: charge transfer resistivity, W: Warburg impedance, R_s_: solution resistivity.

EIS is a non-destructive method to measure impedance (Z) as a function of the alternating current amplitude in a range of frequencies. The usual electrochemical measurements, such as cyclic voltammetry (CV), which uses DC potential, does not provide enough information about the electrochemical reactions that occurred at the interface of an electrode and electrolyte. EIS, which can be performed in a broad range of frequencies in different potentials, gives valuable information about electrical features, such as charge transfer resistivity (R_c_), solution resistivity (R_s_), diffusion resistivity (Warburg impedance), and equivalent electric circuit simulation. In a simple system, the equivalent circuit model is called a Randle circuit (Figure 15).

**Figure 16:**
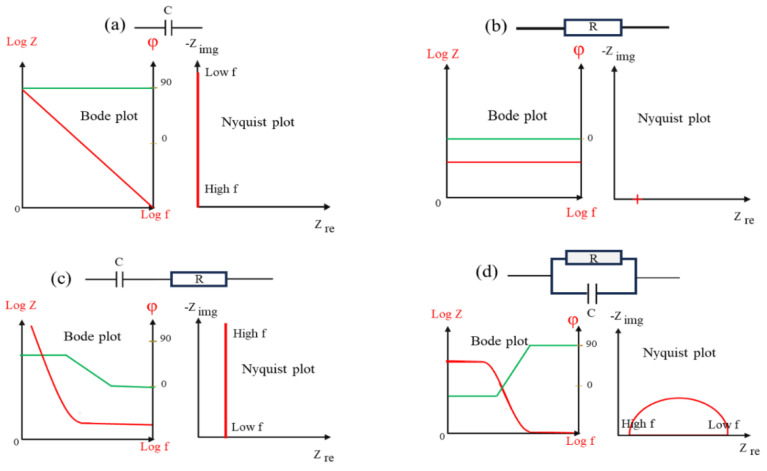
Bode and Nyquist plots representing the essential elements in a circuit that simulates the impedance measured by EIS. These elements are (**a**) a capacitor, (**b**) a resistor, (**c**) a capacitor in series with a resistor, and (**d**) a capacitor in parallel with a resistor. Red curves correspond to left Y axis, and green curves correspond to right Y axis.

In the EIS method, a small AC potential is applied to DC potential. Since no electrochemical reaction is 100% reversible when the applied potential is reversed, using a high amplitude of AC potential can distort the electrochemical stability of the system [186]. To minimize such perturbation caused by AC potential, a very small AC signal is applied on a constant DC potential. The variation of impedance with frequency is often displayed in two ways: a Bode plot and a Nyquist plot (Figure 16).

In a Bode plot, log \Z\ and ϕ are both plotted against log ω, where Z is impedance and ω is frequency. In a Nyquist plot, the imaginary part of impedance (Z_Im_) is plotted versus the real part of impedance (Z_Re_) for different values of ω. Figure 16 shows the most important components in a circuit and the Bode and Nyquist plots representing them.

**Figure 17:**
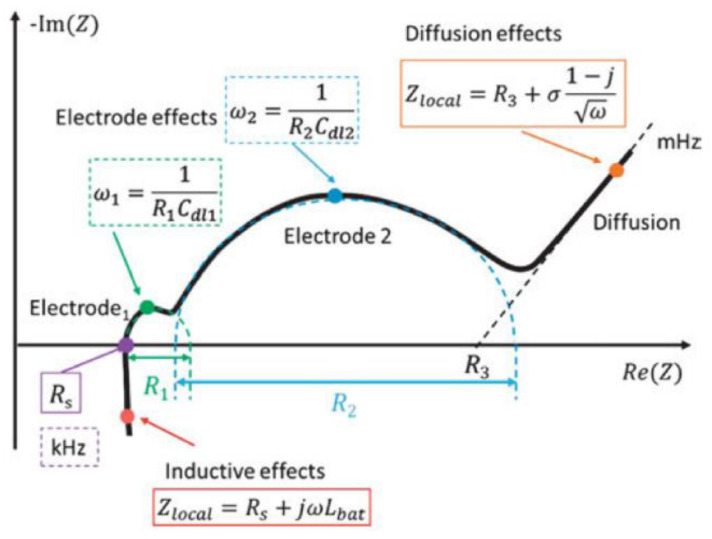
Typical impedance spectrum of a battery in an exaggerated Nyquist plot. Here, *R_s_* is the series resistance, *R*_1_ and *R*_2_ are the charge transfer resistances of the electrodes, *C*_dl1_ and *C*_dl2_ are the double layer capacitances of the electrodes, *σ* is the Warburg coefficient, *R*_3_ is the intersection of the Warburg impedance. Reproduced from Ref. [187], CC BY-NC-ND 4.0.

In a relatively short time of measurement, a Nyquist plot can provide information about the inner state of a system in terms of electrolyte properties, diffusion layer, ion migration, electrode surface property, and electrode reactions (Figure 17). This method is independent of leakage current and fluctuating voltage. At low frequencies, migration phenomena are dominant; at middle frequencies, electrode reaction is dominant; and inductive effect and structure porosity are dominant at high frequencies [187].

Every electrochemical cell has its special phenomena and, consequently, its impedance curve models, which can be expressed analytically with an equivalent circuit consisting of electrical elements such as capacitors, resistors, and inductors. This equivalent circuit can simulate the internal electrical properties of a cell. To run an impedance test, one needs to adjust an appropriate frequency range, dc potential, ad potential, and curve type.

#### 6.1.2. Cyclic Voltammetry

**Figure 18:**
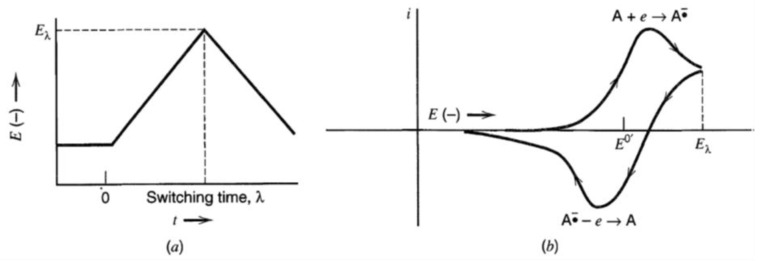
Cyclic voltammetry. (**a**) Cyclic potential sweep, (**b**) resulting cyclic voltammogram. Reproduced from [187] with permission from John Wiley & Sons.

Cyclic voltammetry (CV) is a potential sweep technique frequently used in electrochemical study and it provides abundant information about the redox reactions at an electrode. It is among the most widely practiced electrochemical methods. Figure 18 shows a linear (t, time) complete scan where the backward scan starts when the forward scan reaches the switching potential E_λ_. The potential at any time point is given by [188]:

(0 < *t* ≤ *λ*) *E* = *Ei* − *vt*

(*t* > *λ*) *E* = *Ei* − 2*vλ* + *vt*

where:

*v:* scan rate (v/s);
E_λ_: switching potential;
E_i_: initial potential; and
λ: run time at switching potential.

The peak in the negative scan represents a reduction taking place at the working electrode, and the peak in positive scan shows oxidation.

**Figure 19:**
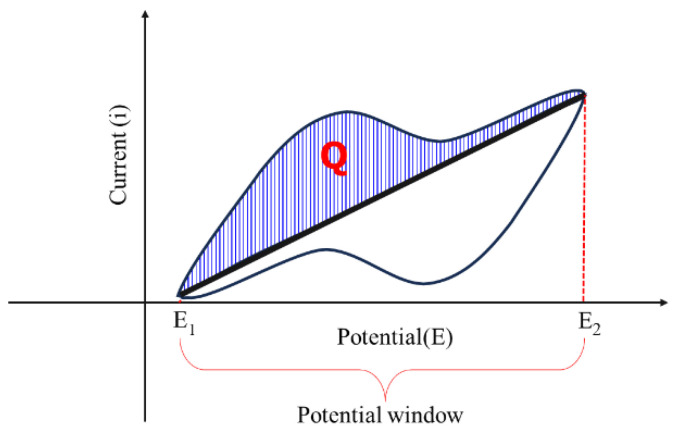
Cyclic voltammogram and determination of capacity.

By this voltammogram, the capacitance of an electrode material can be calculated. The charge (Q) accumulated on the electrode is calculated by the area under the CV curve in one direction from E_1_ to E_2_ (see Figure 19), and the capacitance (C_i_) of the electrode can be calculated based on the equation below:

$$C_{i}=\left|\frac{Q}{E_{2}-E_{1}}\right|$$

#### 6.1.3. Cyclic Charge–Discharge (CCD)

CCD is a technique frequently used to evaluate specific capacitance, power and energy densities, and cycle stability. Normally, a charge–discharge cycle is performed under a constant current in a determined voltage window. A loop of charge–discharge is called a cycle. The capacitance (C) of each cycle is calculated in farad (F) based on the following equation [189]:

$$C=\frac{Q}{V}$$

where Q is the charge in coulomb (C), and V is the voltage window. Both are the function of a cycle.

Q is calculated based on the equation below [190]:

$$Q=\frac{I\times ∆t}{m(or\;A)}$$

where I/m or I/A is current density per weight (A/g) or current density per area (mA/cm^2^), and Δt is discharging time. So, the calculation of C is based on the following equation where ΔV is the voltage window:

$$C_{s}=\frac{I\times ∆t}{m(or\;A)\times ∆V}$$

Energy and power densities of the electrode are calculated based on the following equations:

$$Energy\;density\;(Wh\cdot Kg^{-1})=\frac{1}{2}\;C_{s}∆V^{2}\frac{1000}{3600}$$

$$Power\;density(W\cdot Kg^{-1})=\frac{E}{t_{d}}\times 3600$$

where C_s_ = specific capacitance (F/g or mF/cm^2^); ΔV (V) is the maximum potential window; E is the energy density; and t (s) is the discharging time.

### 6.2. Four-Point Probe for Resistivity Measurement

**Figure 20:**
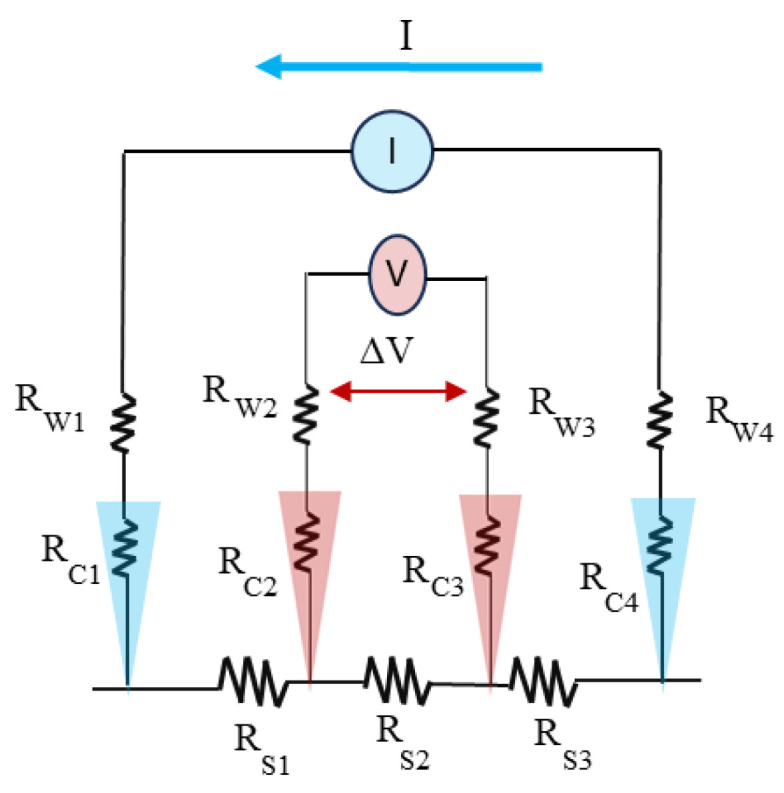
Four-point probe to measure membrane resistivity.

The four-point probe technique is most commonly used to measure surface or sheet resistivity. The set-up consists of four small metal tips lined up with the same separation, as shown in Figure 20. A constant current is applied between the two outer tips, and the voltage drop is measured between the inner tips. The resistivity of the surface is given by the equation below, where C_F_ is the correction factor based on the size of the specimen relative to the dimension of the probe and the ratio of the thickness of the conducting layer to probe separation [191].

$$\sigma =\frac{V}{I}\;C_{F}$$

### 6.3. Infrared Spectroscopy

**Figure 21:**
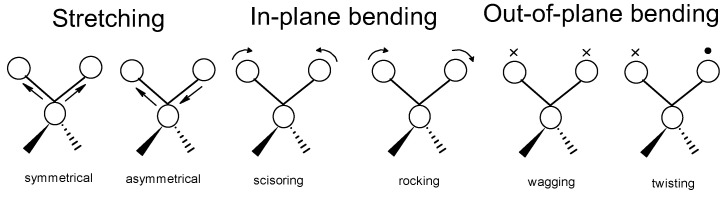
Molecular vibration modes.

Infrared spectroscopy (IR) is a conventional method to study the chemical nature of substances, including polymers. IR is based on the interactions between the molecular structures of a material and the electromagnetic radiation in the infrared range. When IR radiation is in contact with a test material, the chemical bonds of the material absorb IR energy at characteristic frequencies according to the chemical bond vibration modes, such as stretching, bending, and scissoring [192]. The balls represent the atoms connected by chemical bonds that act as springs. Based on how the atoms move, the vibration mode can be determined. The movement of an atom toward or away from other atoms along the line of the spring represents a stretching vibration. Stretching can be either symmetric or asymmetric. A molecule with three or more atoms can experience multiple modes of vibration, as summarized in Figure 21.

**Figure 22:**
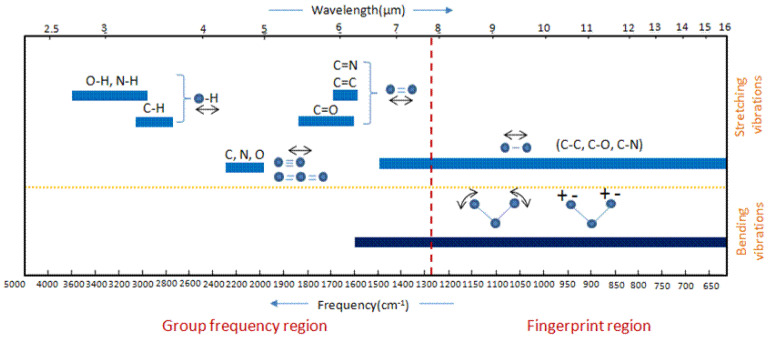
Group frequency and fingerprint regions of the mid-infrared spectrum. Reproduced from [193], CC BY 4.0.

Each chemical group has particular interactions with infrared radiation, which causes energy absorptions at characteristic frequencies that differ, one chemical group from another. The region below 1800 cm^−1^ is the fingerprint of a material, where the peaks carry the most useful information, and can be complicated to interpret. Figure 22 shows the typical infrared absorption positions and intensities of important chemical bonds.

### 6.4. X-ray Photoelectron Spectroscopy (XPS)

**Figure 23:**
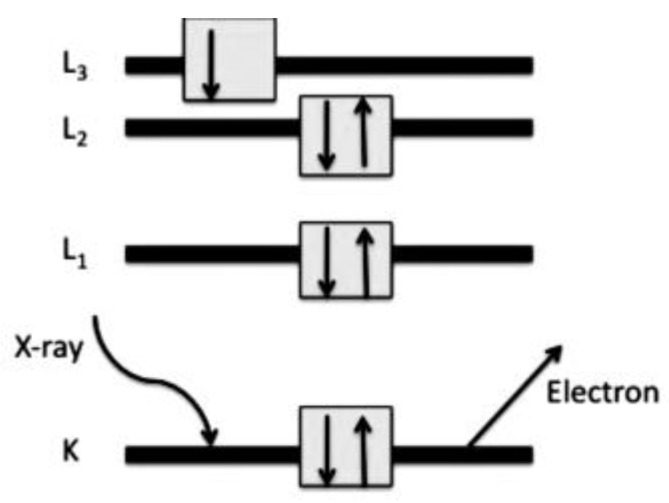
Electron excitation from K-shell’s atoms. Reproduced from [194] with permission from Pavan M. V. Raja & Andrew R. Barron, Rice University (CC BY 4.0).

XPS is a non-destructive surface analysis method that allows identifying the chemical and electronic structures of the top-most layer of a material. It gives information about the electronic valance structures of chemical bonds. The atomic composition of a sample can also be determined by this method. The under-test sample is excited by a high-energy X-ray source. While the X-ray penetration depth into a sample is deep, the free path (λ) of the photoelectrons escaping from the sample is very short, e.g., 5 to 20 Å, leading to a sampling depth of 15 to 60 Å (3λ). By changing the take-off angle, the sampling depth can be further reduced. Consequently, this method gives surface rather than bulk chemical information. In this method, atoms absorb X-ray energy, then an electron from the K-shell, which is the lowest energy shell of the atom, is ejected, and its kinetic energy (KE) is measured. The binding energy (BE) of that photoelectron can be calculated based on the KE and the energy of the incident beam (h*v*) [194]. Figure 23 shows the energy-ejecting mechanism.

Because the BE of photoelectrons is characteristic for each element (except hydrogen), the number of detected electrons with a specific BE is proportional to the number of atoms of the corresponding element in the testing sample, which provide the relative percentage of each element in that sample. Table 5 shows the electron BE and the orbitals from which the electrons are ejected.

### 6.5. Scanning Electron Microscopy (SEM)

In a material surface study, scanning electron microscopy (SEM) is one of the more important methods that can provide useful information about the material surface structure. Compared to optical microscopy, which is the oldest method with a maximum magnification of 1000 times a sample’s original size, SEM works based on electrons, and the magnification of the image can reach up to 300,000 times that of the sample’s original size. SEM is a non-destructive method in which a high-energy beam of electrons is applied to the surface of a sample and penetrates about 1 μm of depth to generate the secondary electrons (SEs) and backscattered electrons (BSEs) that are used to produce an image of sample topography. In order to have a clear, sharp image, parameters such as brightness and intensity can be adjusted by the operator. Low accelerating voltages (less than 5 kV) can provide images with rich surface details; however, high accelerating voltages (15-30 kV) that can penetrate underneath the surface will reflect signals that include some details about the interior of the specimen [195].

## 7. Conclusions

Multiple factors affect the overall performance of electrodes and supercapacitors. Only carefully designed strategies can lead to high capacitance, balanced energy and power densities, and acceptable cyclic stability. Nanostructured CP electrodes provide a high number of redox reaction sites at the electrode/electrolyte interface and, consequently, a high capacitance. Such nanostructures can be formed through special polymerization methods or by coating the nanostructured carbon and metal oxide surfaces. Nanostructures may also improve the cyclic stability of CP electrodes because of less stress in thin-walled structures compared with thick structures that have undergone the same strain. Currently, metal oxides and carbon-based nanomaterials, such as graphene and carbon nanotubes, are the leaders. A Co_3_O_4_ electrode achieved 3560 F/g, while a PPy/CoO electrode recorded 2225 F/g, demonstrating the advantage of combining with metal oxide [196]. To compete with carbon and metal oxide-based supercapacitors, CP electrodes are light, less expensive, capable of being flexible, and environmentally friendly. These advantages make CP-based electrodes and supercapacitors suitable for wearable, disposable, and low-energy-density personal electronics. Supercapacitors made of CP may also be used in medical implants because CPs such as PPy and PEDOT are biocompatible. However, CP-based supercapacitors still need to improve their energy density and cyclic stability. To be used in medical implants, biocompatible electrolytes are mandatory. Industry must identify the best composition and structure based on specific applications. Another challenge is how to form and maintain excellent contact between CP and metallic current collectors, particularly when repeated deformation occurs. Obviously, there is still a gap between the research and application of CP-based supercapacitors, which needs our continuous efforts.

## Figures and Tables

**Table 1:** Important characteristics of different electrochemical energy storage systems [7,33,34].

	Supercapacitors (SC)			Lithium-Ion Battery
	EDLC SC	Pseudo SC	Hybrid SC	
Cycle life	10^6^	10^5^	5 × 10^5^	500
Energy density (Wh⋅kg^−1^)	3–5	10	180	250
Power density (W⋅Kg^−1^)	3 × 10^3^	10^7^	10^3^	100
Operating temperature (°C)	−40 to 65	−40 to 65	−40 to 65	−20 to 60
Self-discharge per month (%)	60	60	Not available	4
Type of electrolyte	Aprotic or protic	Protic	Aprotic	Aprotic

**Table 2:** Typical conducting polymers and structures.

Polymers	Theoretical Structures
Polyacetylene (Pac)	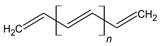
Polypyrrole (PPy)	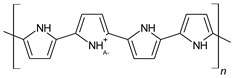
Polythiophene (PTh)	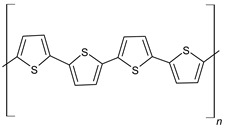
Poly(3,4-ethylene dioxythiophene) (PEDOT)	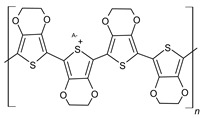
Polyaniline (PANi)	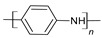
Poly(para-phenylene) (PPP)	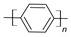
Poly(phenylene-vinylene) (PPV)	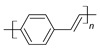
Poly(thienylene-vinylene) (PTV)	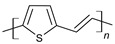
Poly(furylene-vinylene) (PFV)	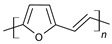
Poly(phenylene sulfide) (PPS)	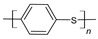
Poly(phenylene-ethylene) (PPE)	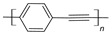
Polyselenophene	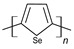
Polyfuran	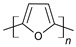
Polyindole	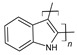
Polyfluorene	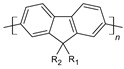
Polypyridine	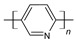
Poly(diphenylamine)	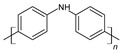
Poly(thieno [3,2-b]pyrrole	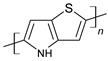

**Table 5:** Some important elemental binding energies. Reproduced from [194] with permission from Pavan M. V. Raja & Andrew R. Barron, Rice University (CC BY 4.0).

Element	Binding Energy (eV)
Carbon (C_1S_)	284.5–285.1
Nitrogen (N_1S_)	396.1–400.5
Oxygen (O_1_s)	526.2–533.5
Silicon (Si_2_p)	98.8–99.5
Sulfur (S_2p3/2_)	164.0–164.3
Iron (Fe_2p3/2_)	706.8–707.2
Gold (Au_4f7/2_)	83.8–84.2

## Data Availability

Not applicable.
